# Successful Control of Heavy Menstrual Bleeding Using High-Intensity Focused Ultrasound Ablation of Fibroid Uterus in a Woman With Eisenmenger Syndrome

**DOI:** 10.7759/cureus.27165

**Published:** 2022-07-23

**Authors:** Vivian W. Y Ng, Vincent Y. T Cheung

**Affiliations:** 1 Department of Obstetrics and Gynaecology, Queen Mary Hospital, The University of Hong Kong, Hong Kong, HKG

**Keywords:** ultrasound-guided, uterine fibroid, high-intensity focused ultrasound, heavy menstrual bleeding, eisenmenger syndrome

## Abstract

Uterine fibroid causes heavy menstrual bleeding and can be difficult to manage particularly in patients with complicated medical history. We present a woman with Eisenmenger syndrome, who presented with heavy menses due to fibroid uterus. She was advised against having hormonal therapy or major surgery under general anesthesia. We successfully controlled her heavy menses and avoided surgery using high-intensity focused ultrasound ablation of her fibroid.

## Introduction

Uterine fibroid is a common gynecological condition that can cause heavy menstrual bleeding. Women can also experience anemia, debilitating pelvic pain, reduced fertility, and pressure symptoms. For many years, surgical management such as myomectomy and hysterectomy has remained to be the standard definitive treatment. However, in recent years, there are more and more women who prefer uterine preservation or wish to avoid major surgery. High-intensity focused ultrasound (HIFU) ablation has emerged as an alternative treatment option [1-3].

## Case presentation

The patient was a 45-year-old nulliparous woman with a history of heavy menstrual bleeding and fibroids for four years. She had ventricular septal defect with Eisenmenger syndrome, pulmonary hypertension pending lung transplant, and ventricular septal defect repair. She also had gout and a history of brain abscess with meningitis requiring burr hole drainage. Her uterus was 16-week size. Due to her complex medical comorbidities, she was advised against the use of hormone therapy and was considered unfit for major surgery under general anaesthesia. 

She had tried tranexamic acid (up to 1000 mg orally four times daily) and a six-month course of gonadotropin-releasing hormone analogue (given by another gynecologist before her referrral) for flow control, and yet she still found her periods debilitating. Her haemoglobin level dropped from 15 g/dL to 10 g/dL over a period of five months. She had persistent iron deficiency anemia despite intravenous iron (iron isomaltoside 1000 mg) and oral iron supplement (ferrous gluconate 300 mg daily). She was thus referred for consideration of HIFU ablation.

**Figure 1:**
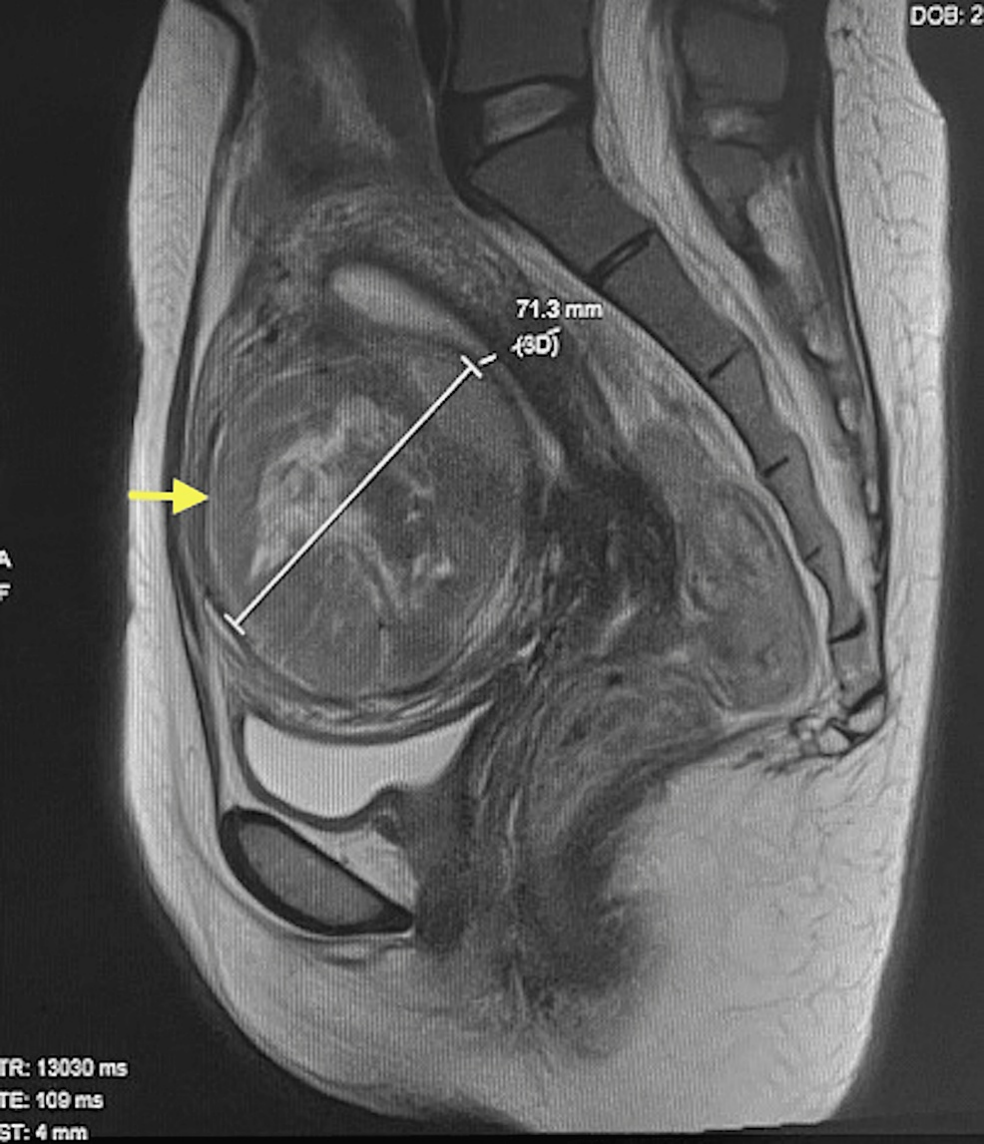
Pre-treatment MRI image Pre-treatment MRI showed an anterior FIGO Type 3 fibroid (arrow).

Magnetic resonance imaging (MRI) showed a solitary anterior intramural fibroid (FIGO Type 3) measuring 7.1x7.7x8.6 cm with heterogenous hyperintensity with central necrosis (Figure 1). She preferred to try HIFU although uterine artery embolization was also offered as an alternative treatment.

Ultrasound-guided HIFU were performed using the JC HIFU system (Chongqing Haifu Technology, Chongqing, China) under monitored anesthesia care. The details of HIFU ablation for fibroids have been described in previous articles published from our institution [2,3]. HIFU was completed after a total treatment time of 77 minutes and a total sonication time of 18.6 minutes. A total energy of 437,483 joules was delivered at an average power of 392 watts (range 350-398 watts). Good greyscale change was observed during treatment. No immediate complications such as skin burn or hematuria was noted. She was discharged home on the next day.

**Figure 2:**
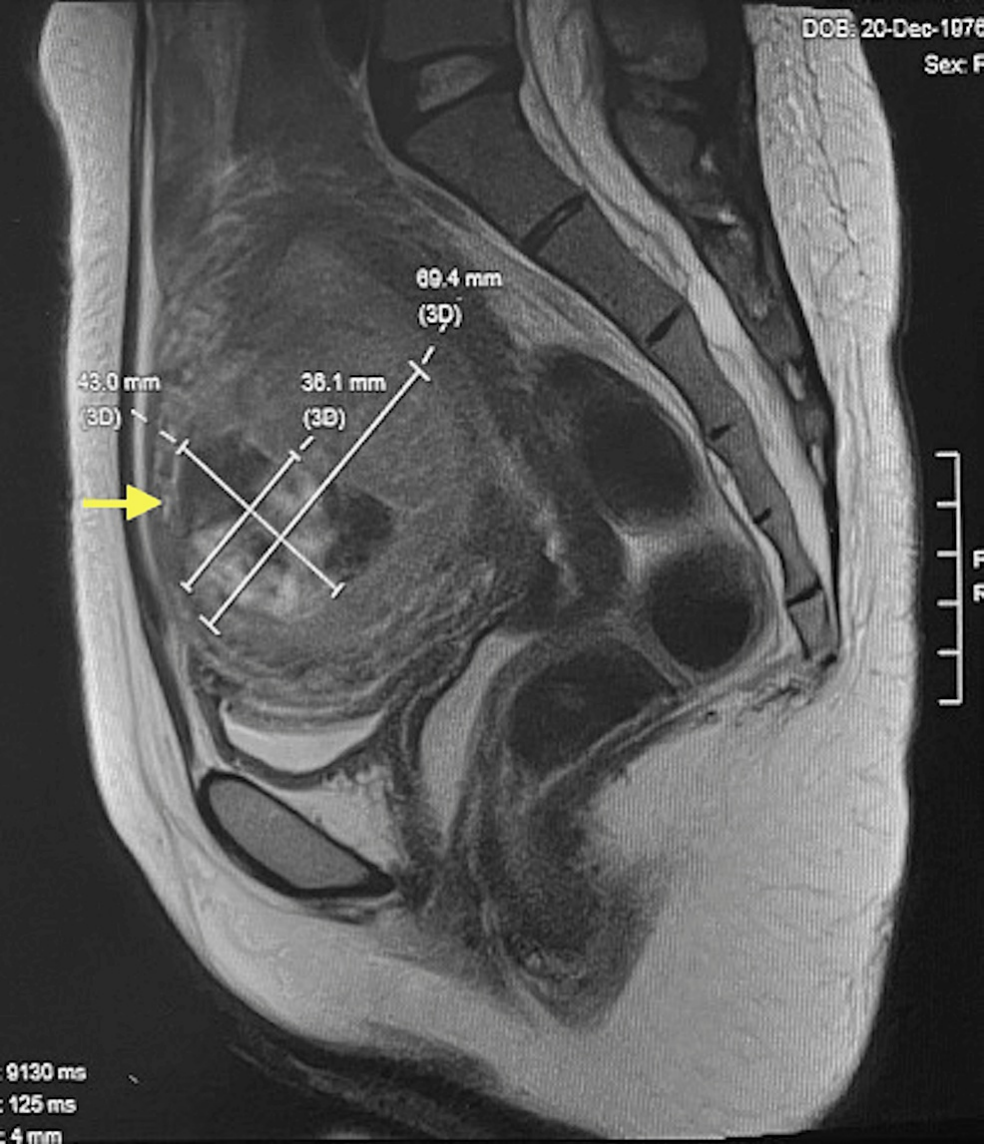
MRI four-month after HIFU HIFU: High-intensity focused ultrasound MRI four-month after HIFU showed a minimally reduced fibroid size (arrow).

MRI four months after HIFU showed that the fibroid measured 6.9x7.5x8.5 cm, which was equivalent to only 6.4% shrinkage of the pre-treatment fibroid volume (Figure 2). A 4.1x4.1x5.4 cm hypo-intense area, which corresponded to a non-perfused volume ratio (NPVR) of 20.6%, was noted. However, despite the minimal shrinkage of fibroid volume and the suboptimal NPVR, the patient reported good symptom control after treatment with her Uterine Fibroid Symptom and Quality of Life (UFS-QoL) questionnaire score improved from the baseline of 32 to 15 at six months after HIFU. Her requirement of tranexamic acid taken orally had significantly reduced from previously 1000 mg four times daily to 500 mg three times daily. Her haemoglobin level was maintained at 14 g/dL. She was satisfied with the treatment response and was advised to continue regular follow-ups at the Gynaecology Clinic.

## Discussion

HIFU employs focused ultrasound waves to generate heat and induce thermocoagulation necrosis at a specific target, without damaging the adjacent tissues. It aims to deliver heat of over 60°C to the target tissue [2,3]. This unique ability has enabled treatment for tumours in the liver, kidney, pancreas, and uterus [4]. The HIFU beam can be guided under magnetic resonance or ultrasound imaging for target localization and treatment monitoring. Ultrasound-guided HIFU, which is less expensive and requires a shorter treatment time than magnetic resonance-guided HIFU, is used in our institution [3,5].

In a review of eight articles published from 2005 to 2012 on ultrasound-guided HIFU ablation of fibroids, 48.2% of patients showed symptom improvement at three months and up to 89.5% at six months after treatment; with a 38.5% and 48.8% reduction of symptom severity scores at 6 and 12 months respectively [6]. The percentages of fibroid volume reduction at three, six, and 12 months were 27.2-47.1%, 47.9-73.1% and 50.3-78.9% respectively [6]. A recent prospective cohort study of 20 patients from our centre also showed a median fibroid volume reduction of 75.9% at 12 months after HIFU. The median UFS-QoL reduction was 44.9% at 12 months; indicating that HIFU could provide sustained relief of fibroid-related symptoms after treatment [3].

Careful patient selection for HIFU ablation ensures treatment success. Patients with pedunculated subserosal fibroids, fibroid suspicious of malignancy, extensive pelvic adhesions such as repeated laparotomies, severe pelvic endometriosis, are generally considered contraindications for treatment [2]. Our patient satisfied the above selection criteria for HIFU treatment. However, a significant area of central necrosis in the fibroid, which this woman had, could be considered a relative contraindication. This was because the necrotic area might interfere with the smooth passage of the HIFU beam, which might result in less response to ablation. On balancing her high risk of anaesthetic and surgical complications, when compared with the relatively minimally invasive nature of HIFU ablation, HIFU was considered to be a much preferred treatment option. 

The non-perfused volume ratio (NPVR) is considered the gold standard for evaluating the effect of HIFU treatment. It refers to the ratio of the non-perfusion volume to the fibroid volume in the MRI, which is done preferably within a week after HIFU ablation. A high NPVR is closely related to treatment effectiveness [1,7-9]. Previous studies have shown that when the NPVR was up to 70%, the two-year treatment effectiveness of HIFU ablation was considered the same as myomectomy [7]. Several factors can affect the NPVR such as fibroid location, vascularity, MRI signal intensity and abdominal wall thickness [7,10]. In our center, MRI for NPVR measurement shortly after HIFU ablation is not routinely performed due to limitation of MRI facilities and our believe that the degree of symptom relief and fibroid volume reduction are more relevant indicators of treatment success [3]. In our patient, the suboptimal NPVR and minimal fibroid shrinkage, even at four months after HIFU ablation, could be explanable by (1) the considerable area of central necrosis could have made the treatment less effective and more difficult to quantify using NPVR; and (2) the concern with her medical comorbidities and the intention to shorten treatment time had resulted in a less extensive ablation. Nonetheless, the improvement of this patient’s symptoms after HIFU had demonstrated the effectiveness of this treatment modality.

## Conclusions

The case illustrates HIFU ablation can be a useful treatment alternative for fibroid uterus in patients with high surgical risks who have failed medical therapy. Patients can have satisfactory symptom relief, despite suboptimal NPVR and fibroid shrinkage, without the need for a major surgery.
